# Induction of Short NFATc1/αA Isoform Interferes with Peripheral B Cell Differentiation

**DOI:** 10.3389/fimmu.2018.00032

**Published:** 2018-01-24

**Authors:** Khalid Muhammad, Ronald Rudolf, Duong Anh Thuy Pham, Stefan Klein-Hessling, Katsuyoshi Takata, Nobuko Matsushita, Volker Ellenrieder, Eisaku Kondo, Edgar Serfling

**Affiliations:** ^1^Department of Molecular Pathology, Institute of Pathology, Comprehensive Cancer Center (CCC) Mainfranken, University of Würzburg, Würzburg, Germany; ^2^Department of Pathology, Graduate School of Medicine, Dentistry and Pharmaceutical Science, Okayama University, Okayama, Japan; ^3^Laboratory of Molecular Biochemistry, School of Life Science, Tokyo University of Pharmacy and Life Science, Hachioji, Tokyo, Japan; ^4^Department of Gastroenterology and Gastrointestinal Oncology, University Medical Center Göttingen, Göttingen, Germany; ^5^Division of Oncological Pathology, Aichi Cancer Center Research Institute, Nagoya, Japan

**Keywords:** B cells, DT40 cells, germinal center, NFATc1, plasmablasts, plasma cells

## Abstract

In lymphocytes, immune receptor signals induce the rapid nuclear translocation of preformed cytosolic NFAT proteins. Along with co-stimulatory signals, persistent immune receptor signals lead to high levels of NFATc1/αA, a short NFATc1 isoform, in effector lymphocytes. Whereas NFATc1 is not expressed in plasma cells, in germinal centers numerous centrocytic B cells express nuclear NFATc1/αA. When overexpressed in chicken DT40 B cells or murine WEHI 231 B cells, NFATc1/αA suppressed their cell death induced by B cell receptor signals and affected the expression of genes controlling the germinal center reaction and plasma cell formation. Among those is the *Prdm1* gene encoding Blimp-1, a key factor of plasma cell formation. By binding to a regulatory DNA element within exon 1 of the *Prdm1* gene, NFATc1/αA suppresses Blimp-1 expression. Since expression of a constitutive active version of NFATc1/αA interfered with *Prdm1* RNA expression, LPS-mediated differentiation of splenic B cells to plasmablasts *in vitro* and reduced immunoglobulin production *in vivo*, one may conclude that NFATc1/αA plays an important role in controlling plasmablast/plasma cell formation.

## Introduction

The maturation of peripheral B lymphocytes is a multistep process. Due to hyper-mutations and class switches in their immunoglobulin (Ig) genes during the germinal center (GC) reaction, B cell maturation results, finally, in the generation of plasma cells that produce large amounts of specific antibodies (Abs) of high affinity, and in memory B cells (1, 2). In contrast to plasma cells that lack a B cell receptor (BCR), memory B cells are able to react immediately onto further infections upon antigen contact through the BCR. Although numerous experimental studies have been devoted to elucidate the generation of plasma B cells and memory B cells, the molecular mechanisms of this important step in differentiation of adaptive immune system remained unclear to a large extent.

In this study, we investigated the role of transcription factor NFATc1 (also designated as NFAT2) in plasma cell formation. NFATc1 is expressed in most of lymphoid cells, but its expression is switched off during plasma cell formation (3). While ablation of NFATc1 did not markedly affect B cell differentiation in bone marrow and periphery, it led to a strong decrease in generation of peritoneal B1 cells (4), and in a mild reduction of BCR-mediated proliferation and increase of BCR-mediated activation-induced cell death (AICD) in B2 cells (5). Surprisingly, in mice bearing NFATc1-deficient B cells no marked defect was observed on the Ab formation upon immunization with NP-KLH, a T cell-dependent antigen, whereas the formation of Abs upon immunization with T cell-independent type II antigen was strongly affected. Moreover, in spite of the marked anti-apoptotic effect of NFATc1 in B cells, the genes targeted by NFATc1 in splenic B cells did not help to explain the phenotype typical for NFATc1-deficient B cells (5). One explanation for this discrepancy could be the opposite effects which is exerted by the various NFATc1 isoforms on AICD and, therefore, on differentiation of lymphocytes (6).

The family of NFAT transcription factors comprises five members. The *Nfatc1* gene belongs to a group of mammalian genes that are expressed in multiple isoforms with opposite functions. Similar to its expression in peripheral T cells (7), due to the use of two alternate promoters (P1 and P2) and poly A sites (pA1 and pA2), and alternate splicing events the *Nfatc1* gene is expressed in six isoforms in peripheral B cells  (5). In splenic B cells persistent signals from the BCR and co-stimulatory receptors lead to the predominant expression of a short isoform, designated as NFATc1/αA, within 24 h. While due to the use of “basal” promoter P2 and of distal pA2 site in resting cells long NFATc1 isoforms are generated, including NFATc1/βC, activation of cells leads to the predominant synthesis of short isoform NFATc1/αA whose synthesis is directed by the proximal pA1 site and promoter P1. The induction of NFATc1/αA is strongly supported by a remote transcriptional enhancer located in the last intron of the *Nfatc1* gene (8). NFATc1/αA lacks the C-terminal peptide of approximately 250 amino acid residues typical for most of the NFATc proteins. This peptide harbors two SUMOylation sites that, therefore, are present in NFATc1/C proteins. When SUMOylated, NFATc1/C was shown to recruit histone deacetylases to and, thereby, suppresses the *Il2* promoter in T cells (9).

The expression of multiple isoforms with antagonistic properties from the same locus suggests that inactivating the entire locus—as in most gene targeting approaches—can lead to misleading results on the functional capacity of the inactivated gene. To circumvent this restriction, we (over-)expressed two individual NFATc1 isoforms, NFATc1/αA and NFATc1/C, in chicken DT40 B cells and murine WEHI 231 pre-B cells. In addition to their marked opposite effect on apoptosis, NFATc1/αA and NFATc1/C exerted a contrary effect on the expression of *Prdm1* gene encoding Blimp-1. Whereas Blimp-1, a key factor of plasma cell differentiation (10), was suppressed by NFATc1/αA, no or a moderate stimulatory effect on Blimp-1 was observed by NFATc1/C. Expression of a constitutive active (ca) version of NFATc1/αA in splenic B cells led to a marked suppression of Blimp-1 expression and plasmablast differentiation. This indicates NFATc1 as an important transcription factor controlling terminal B cell differentiation.

## Materials and Methods

### Mice, Isolation, and Culture of Cells

Animal experiments were performed according to project licenses (Nr.55.2-2531.01-80/10 and 169), which were approved by the Regierung von Unterfranken, Würzburg. If not stated otherwise, 6- to 10-week-old C57BL/6 wild-type (WT) mice were used. *Cd23-cre* mice were described previously (11). Transgenic (tg) *caNFATc1/*α*A* mice express a mutated, ca copy of NFATc1/αA from the *Rosa 26* locus upon cre-mediated removal of a “floxed” STOP sequence (12). Chicken DT40 B lymphoma cells were cultured at 39.5°C with 5% CO_2_ using RPMI-1640 medium supplemented with 10% FCS, 1% chicken serum, 2-mercaptoethanol (50 µM), and l-glutamine (2 mM)  (13). Murine WEHI 231 cells, EL-4 thymoma cells, human Jurkat T leukemia cells and 293 HEK cells were maintained in RPMI-1640 containing 10% FCS at 37°C in 5% CO_2_. Splenic B cells were isolated using Miltenyi’s B cell isolation kit, cultured in X-vivo 15 medium (Lonza) and stimulated as described (5).

### Inactivation of the Chicken *NFATC1* Gene

Segments from the chicken genomic *NFATC1* locus were amplified using PCR primers and subcloned to generate the left and right arms of target vectors. *NFATC1* targeting vectors were constructed by replacing a ~3.3 kb genomic fragment encoding exons 4 and 5 with drug resistance gene cassettes. The targeting vectors were introduced into WT DT40 cells by electroporation, and cloning of the targeted cells was performed by culturing of cells in the presence of blasticidin, histidinol D, or puromycin as described (13).

### Southern Blotting

Two micrograms of genomic DT40 DNA were digested by Sac I, fractionated on a 0.7% agarose gel and transferred to a Hybond N + nylon membrane (Amersham Biosciences, Buckighamshare, UK). The membrane was hybridized with a 600 bp FITC-labeled PCR-amplified genomic fragment from intron 7 of the chicken *NFATC1* gene as probe.

### Generation of WEHI 231 B Cells Expressing NFATc1-Bio Proteins

Full-length murine NFATc1/αA (gi:255759918 in NCBI database) and NFATc1/βC cDNAs (gi: 255759924) were amplified, fused to a bio/avidin-tag (14) and ligated into the retroviral expression vector pEGZ (15). The retroviral pMSCV-F-BirA vector was purchased from BCCM™/LMBP (Gent-Zwijnaarde, Belgium). Retroviral particles were obtained after transfection of retroviral vectors, along with the retroviral packaging plasmids pHIT60 and pHIT123, into HEK 293T cells. After infection, WEHI cells were kept under selective conditions (using zeocin or puromycin) for 14 days. Positive integration and expression of NFATc1/αA-bio, NFATc1/βC-bio and/or BirA constructs was determined by intracellular streptavidin–fluorophore labeling and flow cytometry.

### Real-Time and Semi-Quantitative PCR Assays

Total RNA was isolated from DT40 B cells using the QIAamp RNA Blood kit (QIAGEN). cDNAs were synthesized from 2 µg of total RNA by using oligo dT primers and superscript reverse transcriptase (Invitrogen) and amplified by LA-Taq polymerase (Takara Bio Co. Ltd., Tokyo, Japan). To confirm the inactivation of *NFATC1* gene, primers for the detection of chicken NFATC1 RNA were designed as follows:

Fw chNFATc1, exon3: 5′-GGACCTTATGAACTGCGTATTGAAGTGCAG-3′,
Fw chNFATc1, exon 4; 5′-CATGGTTACTTAGAAAGCGAGCC-3′.
Rv chNFATc1, exon 6; 5′-CTTTCGAGTCTTGCAGAAAGTTATGGCCAG-3′.

For detecting GAPDH3, the probe was labeled with the VIC reporter dye at the 5′-terminal nucleotide and the TAMRA quencher dye at the 3′-terminal nucleotide. Detection of fluorescence during the thermal cycling process was performed using the ABI Prism 7700 Sequence Detection System (ABI/PE).

For detecting *Prdm1* expression RNA was extracted from splenic B cells using TRIzol reagent (Invitrogen) followed by cDNA synthesis with the iScript II Kit (BioRad). Real-time PCR assays were carried out with an ABI Prism 7700 detection system using *Prdm1* and *β-actin* primers sequences as described previously (16).

### Cloning of Human NFATc1 Isoforms and Generation of Expression Vectors

To generate the human *NFATC1* expression vectors carrying a *neo* resistant gene cassette, full-length cDNAs encoding human *NFATC1/*α*A* (or *NFATC1/*α*C*) was subcloned into the pcDNA3.1 vector between its BamHI-KpnI sites by PCR using cDNA of human BALM-14 cells as template. The primers for subcloning the NFATc1/αA and c1/αC isoforms were the following:

c1/αA; 5′-primer: 5′-CGCGGATCCATGCCAAGCACCAGCTTTCCA-3′(+*BamHI* site),
c1/αA 3′-primer: 5′-CGCGGTACCTTAGAAAAAGCACCCCACGC-3′ (+*KpnI* site),
c1/αC; 5′-primer: 5′-CGCGGATCCATGACGGGGCTGGAGGACCAG-3 “(+*BamHI* site), c1/αC, 3′-primer: 5′-CGCGGTACCCTAGGAGTGGGTGCTCGTGC-3” (+*KpnI* site).

For constructing vectors expressing chimeric EGFP-human NFATc1/αA or c1/αC proteins, a complete fragment of EGFP was PCR-amplified from the pEGFP-C3 vector (Clontech, Palo Alto, CA, USA) using the following primers:

5′-primer: 5′-CGCAAGCTTATGGTGAGCAAGGGC-3′ (+HindIII site), and
3′-primer, 5′-CGCGGATCCTCACTTGTACAGCTCGTC-3′ (+BamHI site).

### Apoptosis Assays and Flow Cytometry

Apoptosis assays were done either with the Cy-3 annexin V staining kit (MBL Co. Ltd., Nagoya, Japan) following the manufacturer’s instructions or by stainings with annexin V-APC (BD Sciences) followed by flow cytometry (FACScan, BD Sciences). 3 × 10^4^ cells of each sample were incubated for 20–30 h with 0–50 µg/ml of anti-chicken IgM mAb M4 (#8300-01, SouthernBiotech, Birmingham, AL, USA), and annexin-V-positive cells were detected by flow cytometry. For apoptosis induction by ionomycin, 10^5^ DT40 cells were incubated with 1 µM ionomycin for 18–30 h.

### RNA Expression Profiling

Upon stimulation with 25 µg/ml of M4 anti-chicken IgM mAb M4 Ab (SouthernBiotech) for 6 h, 5 µg of total RNA was used for the preparation of Cy5-labeled cDNAs from each RNA sample. They were hybridized to Filgen’s chicken 18k arrays that contain oligonucleotide probes of 18,000 identified chicken genes (Filgen, Nagoya, Japan). After hybridization, the signals of arrays were red by GenePix 4000B to obtain fluorescence images, and signal intensity of each spot was calculated using Filgen’s software.

### RNA-Seq Assays

Unstimulated WEHI 231 B cells or WEHI cells stimulated for 6, 24, and 96 h by αIgM were deep-frozen in liquid nitrogen. Their RNA was extracted using Qiagen’s RNeasy kit and Illumina’s purification beads. RNA-Seq libraries for next generation sequencing were prepared using 600 ng starting material and Illumina’s TruSeq RNA sample prep kit V2 following the manufacturer’s instructions. RNA sequencing assays were described previously (17). The data were loaded in ArrayExpress (accession no. E-MTAB-4665).

### Immunoblots

Whole cell protein extracts were resolved on 8–10% SDS polyacrylamide gels. Proteins were transferred to nitrocellulose membranes and probed with the mAb 7A6 (sc-7294, Santa Cruz Biotech. Inc., CA, USA) or a polyclonal Ab (#0102-50, Immunoglobe, Himmelstadt, Germany) raised against all isoforms of human NFATc1. For the detection of NFATc1 proteins tagged with a myc epitope and expressed upon transfection into DT40 cells, the mAb 9E10 was used raised against an epitope of c-Myc (Thermofisher, Scientific). As loading control, filters were incubated with actin mAb (CHEMICON International, Temecula, CA, USA) or stained by ponceau red. Signals were developed by chemiluminescence detection using Super Signal™ (Pierce Chemical Company, CA, USA).

### Electrophoretic Mobility Shift Assays (EMSAs)

Nuclear proteins from murine splenic B cells or DT40 B cells were prepared for EMSAs as described previously (7). For detecting NFAT binding, the distal NFAT site from the murine *Il2* promoter was used as probe.

### Chromatin Immuno Precipitation (ChIP)

Chromatin immuno precipitation assays were performed as described (18, 19) with slight modifications. In brief, 5 × 10^7^ WEHI-231 cells were fixed, the reaction was quenched and washed cells were re-suspended in 1 ml swelling buffer on ice for 30 min. Upon adding 40 µl of 10% NP-40, cells were passed through 8× through 21 G needles, nuclei were collected and re-suspended in 0.5 ml sonication buffer. Chromatin was sheared for 10 min (30 s pulses; 35% amplitude) on ice using an ultrasonic-disintegrator (Sonicor). DNA extracts of supernatants were checked for fragment sizes and quantified using a NanoDrop device (ThermoScientific). Chromatin was pre-cleared with 3 µg unrelated Ig Ab (CellSignaling, #2729p) and 40 µl immobilized protein G beads (ImmunoPure^®^ PIERCE, 50% slurry saturated with salmon sperm DNA, #16-157, Upstate; and 2% gelatin from cold water fish skin (FGEL), Sigma-Aldrich, #G7765). Chromatin in 300 µl sonication buffer was incubated with 25 µl of streptavidin agarose resin (ThermoScientific, #20347; 50% slurry saturated with Salmon Sperm DNA and FGEL), or 2 µl NFATc1 Ab (7A6, BD 556609), or 2 µl Ig Ab, or 2 µl H3 Ab (CellSignaling; #4620s), or 2 µl H3K9me3 Ab (abcam ab8898), or 2 µl H3K9ac Ab (Merck 07-352) at 4°C o.n. Samples with Abs were additionally incubated with saturated protein G beads for 1 h. All beads were washed, and chromatin complexes were eluted twice by incubation with 250 µl of elution buffer. Eluates were supplemented with 21 µl 5 M NaCl and 2 µl RNase (10 mg/ml) for removal of cross-links. DNA was extracted for PCR assays using the following primers:

*Il2* F CCTGTGTGCAATTAGCTCA
*Il2* R CTCTTCTGATGACTCTCTGGA
*Rcan1* F GCGGCATAGTTCCCACTGGTA
*Rcan1* R GAGTGCTGGGCTTTTCATCCA
*Ppp3ca* F TTTTCTTCAGGGTGCCAGTC
*Ppp3ca* R CAGTCTGTTCCCATCCACCT
*Prdm1* F AAACGTGTGGGTACGACCTT
*Prdm1* R AGCGCTGGTTTCTACTGAGG
β*-actin* F CGGTTCCGATGCCCTGAGGCTCTT
β*-actin* R CGTCACACTTCATGATGGAATTG
*Prdm1* promoter F TATCTGCCACTTCCTCTTTC
*Prdm1* promoter R ATGCAAATCTTCTCTGCTGT

### Immunizations of Mice and ELISAs

Age-matched mice (of 8–10 weeks) were immunized with NP-KLH (100 μg/mouse, Alum precipitated; i.p.). NP-specific Ab levels were determined in sera of mice immunized with NP-(27)-KLH for 21 days (5). Samples were applied in threefold serial dilutions starting from a 1:20 initial dilution, and Ig concentrations were quantified against a reference serum by ELISA. NP-specific Abs were determined using MaxiSorp plates (Nunc) coated with 10 µg/ml NP-BSA. Isotype-specific Abs coupled to alkaline phosphatase were used for detection according to manufacturer’s instructions (SouthernBiotech).

### Immunohistochemistry of Human Tonsils

Informed consents were obtained from patients for this study. Sections of formalin-fixed tonsils embedded in paraffin were automatically stained on a Ventana Bench Mark XT (Roche Diagnostics, Japan) using the *ultra view* DAB and/or Alp-Red detection system. Rabbit polyclonal Ab for NFATc1/α and mAbs for CD20, CD79a, and CD35 were used for double stains, 7A6 mAb was used in combination with αIgM polyclonal Ab. Alexa 488-goat αmouse IgG (green) and Cy3-goat αrabbit IgG (red) were used for double immunofluorescence.

### Statistical Analysis

Statistical analyses were performed using GraphPad (Prism) software, version 6.0. Data are presented as mean ± SEM. Unpaired *t*-tests were performed to evaluate the statistical significance. Statistical significances were calculated and indicated (***p < 0.001, **p < 0.005, and * *p* < 0.05).

## Results

### NFATc1 Induction in Murine Splenic B Cells

**Figure 1:**
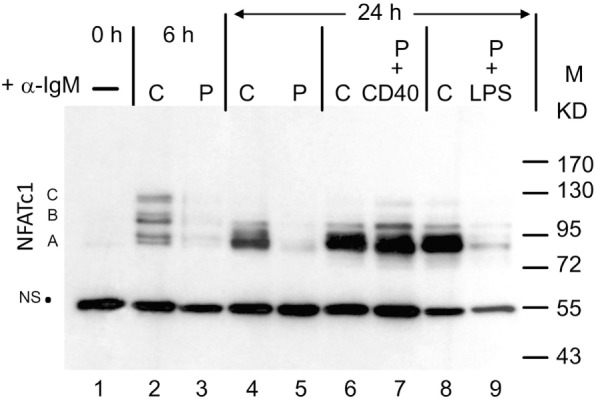
Induction of NFATc1 proteins in splenic B cells. Western blot using the 7A6 mAb directed against all NFATc1 proteins. B cells were left untreated (−), continuously treated (C) with α-IgM for 6 or 24 h, or “pulsed” (P) for 30 min at 4°C with α-IgM, washed and incubated for 4 h without or with α-CD40 or LPS. M, molecular weight marker; NS, non-specific band that indicates—as the ponceau red stain of the filter (not shown)—equal loading of the gel. One typical blot of more than three assays is shown. A, B, and C, NFATc1 isoforms.

When whole protein extracts from freshly prepared murine splenic B cells cultured *in vitro* were analyzed for NFATc1 induction in Western blots, six protein bands were detected upon α-IgM stimulation for 6 h *in vitro*. These bands correspond to the six prominent NFATc1 isoforms described previously for murine and human T cells (6). Extension of α-IgM stimulation to 24 h resulted in the predominant generation of short isoform NFATc1/αA (Figure 1). A similar effect was observed upon adding Abs directed against CD40 (α-CD40) to B cells that were “pulsed” for 0.5 h by α-IgM at 4°C, washed and maintained in culture for 24 h. “Pulsed” B cells stimulated by α-IgM for 0.5 h alone revealed very low NFATc1 levels, even after washing of cells and a further incubation for 24 h in culture. However, the addition of α-CD40 to the α-IgM-“pulsed” led to strong NFATc1/αA induction, whereas addition of LPS exerted a relative mild effect (Figure 1). These observations suggest that the BCR and co-stimulatory signals lead to the accumulation of short isoform NFATc1/αA that represents the most prominent nuclear NFAT protein in effector B cells (16).

### Human NFATc1/αA Protects Chicken DT40 B Cells against Cell Death

To investigate the functional relevance of NFATc1/αA induction in B cells, we expressed in chicken DT40 B cells human NFATc1/αA upon inactivation of endogenous chicken *NFATC1* gene. Due to the existence of three *NFATC1* alleles in DT40 cells, three rounds of gene targeting had to be performed by replacing exons 4 and 5 of the chicken *NFATC1* gene with gene cassettes for selection. This targeting strategy resulted in the ablation of *NFATC1* transcription (Figures S1A–C in Supplementary Material). When we introduced a cDNA encoding either human NFATc1/αA or the long isoform NFATc1/αC both NFATc1 proteins were properly expressed. Upon stimulation with chicken α-IgM (M4) or ionomycin, the human NFATc1 proteins were rapidly translocated into the nuclei of chicken cells (Figures S2A–D in Supplementary Material). Testing the DNA binding activity of NFAT1/αA in EMSAs using nuclear proteins from untreated and *NFATC1^−/−/^*^α^*^A^* DT40 cells treated by TPA and ionomycin (T+I) for 4 h, we observed a strong increase in NFAT binding to the distal NFAT binding site from the murine *Il2* promoter. Transfection of an NFAT-directed luciferase reporter gene into *NFATC1^−/−/^*^α^*^A^* DT40 cells showed a moderate 5- to 10-fold increase in NFAT activity compared to *NFATC1^−/−/−^* cells whereas about the same NFAT activity was measured in WT and *NFATC1^−/−/^*^α^*^A^* cells (Figures S2E,F in Supplementary Material).

**Figure 2:**
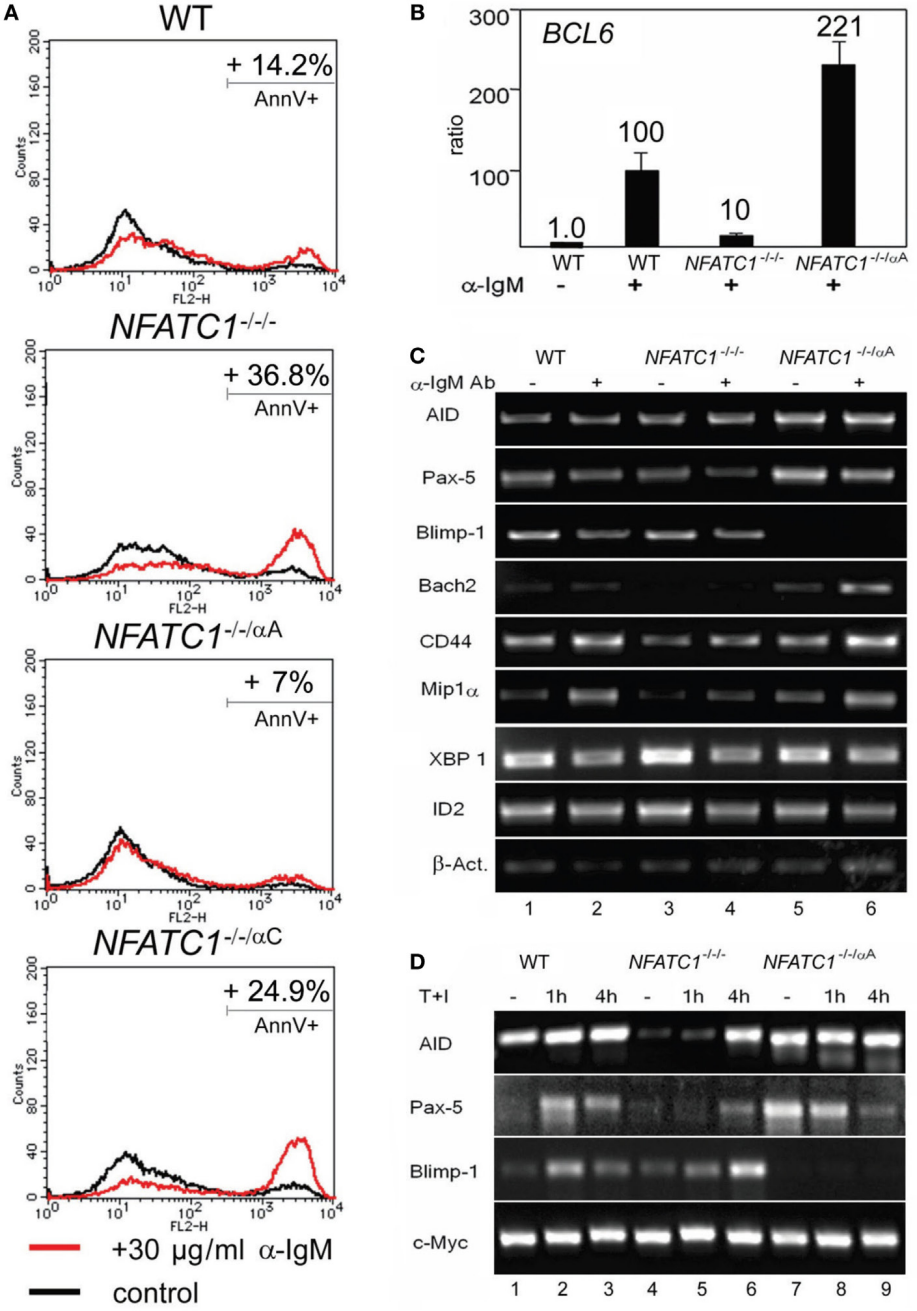
NFATc1/αA suppresses cell death and *Prdm1/*Blimp-1 expression in chicken DT40 B cells. **(A)** WT, *NFATC1^−/−/−^, NFATC1^−/−/^*^α^*^A^*, and *NFATC1^−/−/^*^α^*^C^* DT40 cells were stimulated with 30 µg/ml α-IgM M4 Ab for 28 h. Cells were stained by annexin V-Cy3. Shown is the percentage of apoptotic cells in one typical experiment from more than three. **(B)** Real-time qPCR assays. RNAs were isolated from either untreated DT40 B cells—or from cells treated with 30 µg/ml αIgM for 6 h (+) using a QIAamp RNA Blood kit (QIAGEN), and quantitative RT-PCR assays were performed using TaqMan probes for detecting Bcl-6 and GAPDH3 RNAs as internal control. **(C,D)** Human NFATc1/αA affects the gene signature of DT40 cells. RNAs from untreated DT40 B cells—or from cells treated with α-IgM for 4 h **(C)** or T+I for 1 and 4 h **(D)** were assayed in semi-quantitative PCR assays. In **(C,D)**, one typical assay from two reactions is shown.

Stimulation of DT40 cells by chicken α-IgM rapidly induces apoptosis (20). Using 30 µg/ml α-IgM for 28 h, we observed approximately 14% of annexin V-positive, apoptotic cells, in DT40 cells compared to 3% of apoptotic cells in non-stimulated cells. Stimulation of *NFATC1^−/−/−^* cells led to apoptosis in approximately 37% of cells whereas *NFATC1^−/−/^*^α^*^A^* cells showed a marked protection against AICD. Almost no protection was observed for *NFATC1^−/−/^*^α*C*^ cells expressing the long NFATc1 isoform (Figure 2A). To identify NFATc1 target genes controlling the apoptosis of DT40 cells, we hybridized cDNA probes generated from DT40 RNAs to oligonucleotide arrays which represented 18,000 chicken genes (Filgen, Nagoya, Japan). Among the genes whose expression was strongly enhanced in α-IgM-induced *NFATC1^−/−/^*^α^*^A^* cells were the *BAG2* and *BCL6* genes. Bag-2 is a co-chaperone which, by binding to Bcl-2, could support the anti-apoptotic activity of Bcl-2 (21). Bcl-6 is highly expressed in GC B cells in which, among other effects, it suppresses cells from apoptosis induced by DNA damage (22). In real-time PCR assays using TaqMan probes, we detected a more than 20-fold increase in expression of *BCL6* RNA between *NFATC1^−/−/−^* and *NFATC1^−/−/^*^α^*^A^* cells (Figure 2B). When we investigated the expression of Bcl-6 target genes in semi-quantitative PCR assays in non-stimulated DT40 cells, or in cells stimulated by α-IgM we observed a suppression of Blimp-1 expression and upregulation of Bach-2 in *NFATC1^−/−/^*^α^*^A^* cells, particularly upon stimulation (Figure 2C). Bach2 is a transcriptional repressor which is highly expressed in mature B cells, controls the GC reaction (23) and, along with Bcl-6, represses *Prdm1*/Blimp-1 gene expression (24). However, similar to the *XBP1* gene that encodes a transcription factor required for plasma cell differentiation (25), the RNA levels of further Bcl-6 target genes, such as of *CD44, ID2*, and *CCL3*/Mip1α genes (26), remained unaffected (Figure 2C).

In a further set of PCR assays using RNAs from DT40 B cells treated with T + I for 1 and 4 h, we observed also a defective AID and Pax-5 expression in *NFATC1^−/−/−^* DT40 B cells, compared to WT cells, and again a marked suppression of Blimp-1 expression in *NFATC1^−/−/^*^α^*^A^* cells overexpressing NFATc1/αA (Figure 2D). These data suggest an opposite effect of NFATc1/αA on the GC reaction and plasma cell formation.

### NFATc1/αA Affects Gene Expression in Murine WEHI 231 B Lymphoma Cells

**Figure 3:**
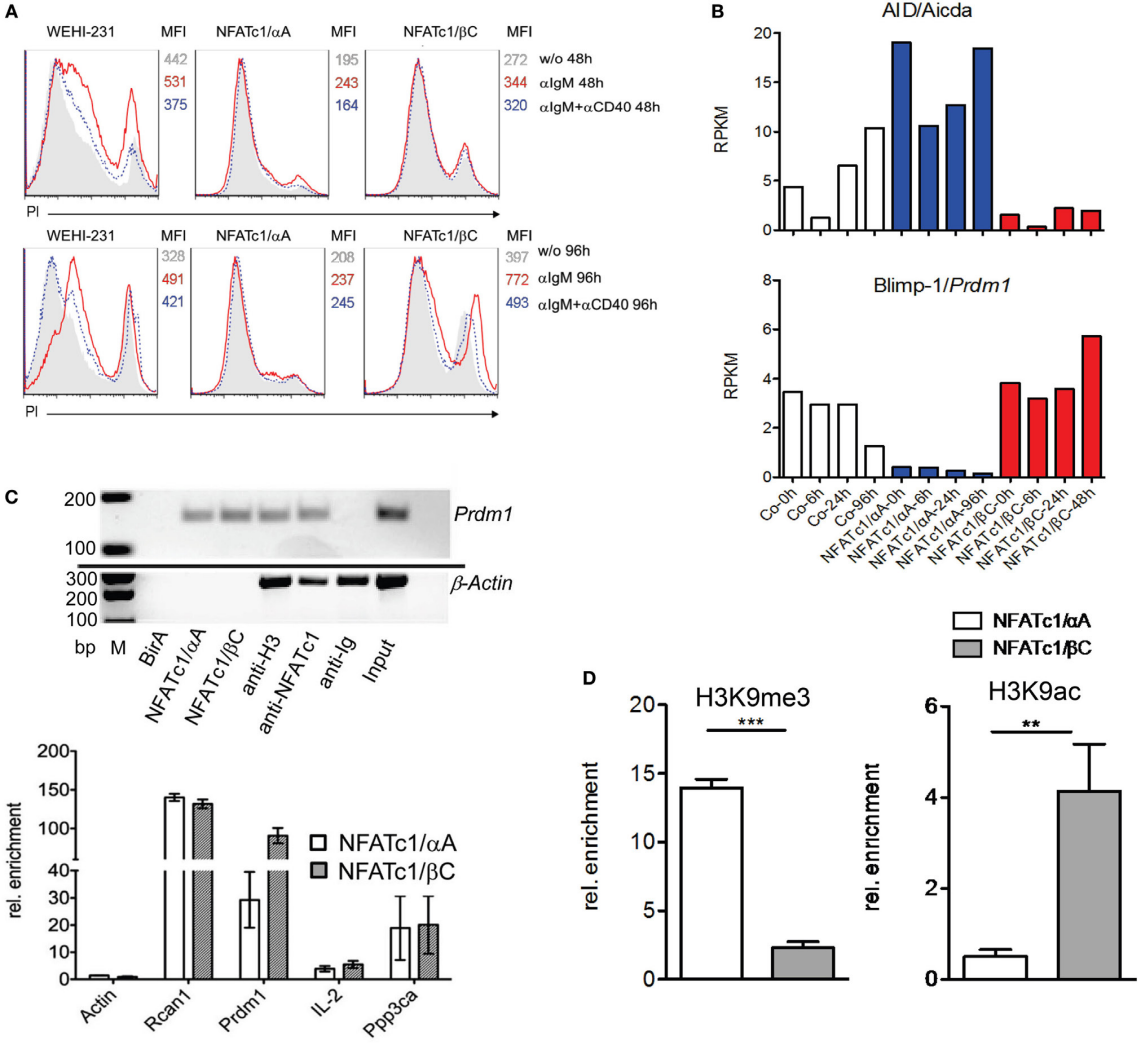
Effect of NFATc1/αA-bio and NFATc1/βC-bio proteins on cell death and the expression of *Aicda* and *Prdm1* genes in murine WEHI 231 B lymphoma cells. **(A)** WEHI 231 B cells stably infected with retroviral vectors expressing BirA (WEHI-231), Bir A and NFATc1/αA-bio (NFATc1/αA), or BirA and NFATc1/βC-bio (NFATc1/βC) were stimulated with α-IgM or α-IgM α-IgM + αCD40 for 48 or 96 h. Apoptosis was determined by PI staining. MFI: Mean fluorescence intensity. **(B)** Wild-type (WT) WEHI cells (Co) or cells expressing NFATc1/αA-bio (blue) or NFATc1/βC-bio (red) were left unstimulated or stimulated for 6, 24, or 96 h with α-IgM. RNA was isolated and converted to cDNA libraries. DNA stretches of 50 bp were sequenced on a Illumina HiSeq2500 platform using the Truseq SBS kit-HS V3. Shown are the RNA reads (RPKM) from the *Aicda* and *Prdm1* genes in the three types of WEHI cells. Results of one from two assays are shown. **(C)** Chromatin immuno precipitation (ChIP) assays for the binding of NFATc1-bio proteins to the *Prdm1* gene in WEHI cells stimulated with T+I for 6 h. In the upper panel semi-quantitative PCR assays are shown for the detection of *Prdm1* (and β*-Actin*) DNA in chromatin precipitations. In the first three lanes, chromatin from WEHI cells transfected with BirA alone, with NFATc1/αA-bio (+BirA) or NFATc1/βC-bio (+BirA) was precipitated with streptavidin-agarose beads. In the next lanes, chromatin was precipitated with Abs specific for histone H3, NFATc1 (7A6), and immunoglobulin. In the last two lanes, DNA input and H_2_O controls are shown. One typical assay from three assays is shown. In the lower panel the enrichment of β*-Actin, Rcan1, Prdm1, Il2*, and *Ppp3ca* DNAs precipitated with streptavidin beads from WEHI cells expressing either NFATc1/αA-bio or NFATc1/βC-bio is shown. Mean values of three assays are shown. **(D)** ChIP assays indicating histone modifications at the *Prdm1* promoter. ChIP assays were performed with chromatin from WEHI cells overexpressing NFATc1/αA-bio (+BirA) (open bars) or NFATc1/βC-bio (+BirA) proteins (gray bars) using Abs directed the histone modifications H3K9me3 and H3K9ac, respectively. In semi-quantitative PCR assays, primers detecting the *Prdm1* promoter (28) were used. Mean values of three assays are shown.

To extend the findings obtained for chicken B cells to mammalian B cells, we studied murine WEHI 231 B cells stably transfected with vectors expressing the biotin ligase BirA from *Escherichia coli*, and either bio-tagged NFATc1/αA-bio or NFATc1/βC-bio. Due to the co-expression of BirA in these cells, bio-tagged proteins are biotinylated and can be precipitated with streptavidin beads (27). Both NFATc1-bio proteins were properly expressed in WEHI cells (Figure S3 in Supplementary Material) and translocated into the nucleus upon α-IgM stimulation (17). Similar to DT40 NFATc1*^−^*^/^*^−^*^/αA^ B cells, NFATc1/αA-bio expressing WEHI cells were protected against α-IgM-mediated AICD (Figure 3A). Stimulation of WT and NFATc1/βC WEHI cells with α-IgM or αIgM + αCD40 for 48 or 96 h led to a significant increase in cell death compared to cells (over-) expressing NFATc1/αA (Figure 3A).

Using RNA from WEHI cells stably transfected with NFAT-expressing vectors for genome-wide RNA-Seq assays, we detected more than 2,000 genes whose RNA levels were changed more than twofold by NFATc1/αA-bio, and somewhat less genes whose RNAs were either affected by NFATc1/βC-bio, or by both NFATc1-bio proteins. Among the genes affected by both NFAT factors were the *Aicda* and *Prdm1* genes encoding AID and Blimp-1, respectively. While NFATc1/αA-bio enhanced *Aicda* expression 2- to 5-fold, it suppressed almost 10-fold *Prdm1* transcription in WEHI cells. By contrast, NFATc1/βC-bio suppressed *Aicda* expression and exerted a moderate stimulatory effect on the transcription of *Prdm1* gene (Figure 3B). Using the same cells in ChIP assays, we observed the specific binding of both NFATc1/αA-bio or βC-bio to a regulatory site within exon 1 of *Prdm1* gene which was described to be highly conserved between mouse and human (29). In real-time PCR assays, we determined a 100-fold enrichment of NFATc1/βC-bio and a 30-fold enrichment of NFATc1/αA-bio binding to this site, compared to a control site from the β*-actin* gene. This is comparable to the binding of NFATc1-bio proteins to *Rcan1*, a well-known NFATc1 target gene in B cells (5), whereas a markedly weaker binding was detected to the *Ppp3ca* gene encoding calcineurin’s catalytic A subunit, and the *Il2* gene that is inactive in B cells. No NFATc1-bio binding to the *Prdm1* gene was detected in WEHI cells expressing BirA alone, and no specific precipitation of β-actin chromatin was observed with the NFATc1 mAb 7A6 which has frequently been used in ChIP assays (Figure 3C).

The expression of the NFATc1 isoforms and their effect on *Prdm1* gene expression correlated well with the occurrence of specific histone modifications at the *Prdm1* promoter. While in WEHI cells overexpressing NFATc1/αA a strong increase in H3K9me3, a mark for inactive heterochromatin (30), was detected, a decrease was observed for H3K9ac, a mark for active transcription (31). Opposite chromatin modifications were determined for the *Prdm1* promoter in WEHI cells overexpressing NFATc1/βC (Figure 3D).

### NFATc1/αA Suppresses Plasmablast Formation and Ab Production

**Figure 4:**
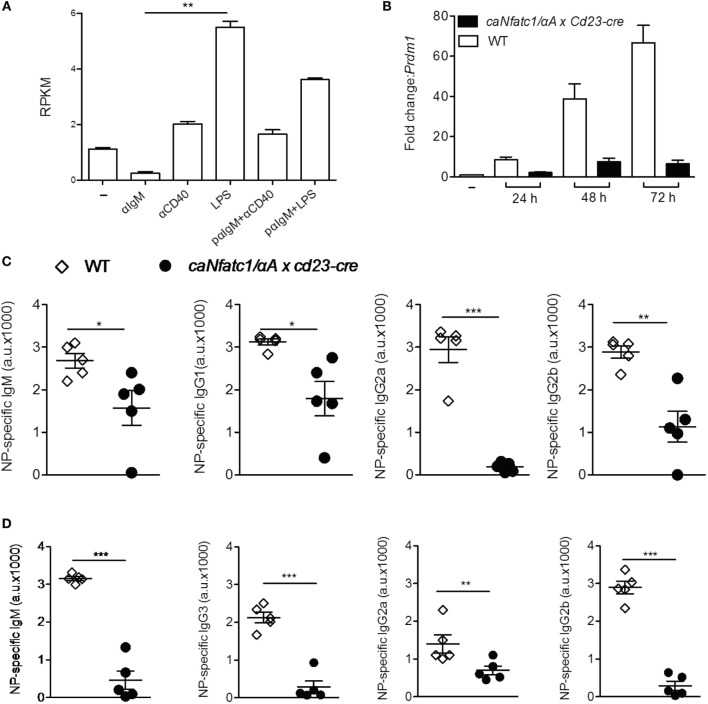
Suppression of plasmablast differentiation and of IgG class switch by caNFATc1/αA. **(A)** Changes in *Prdm1* RNA levels in splenic B cells upon stimulation with α-IgM, α-CD40, or LPS for 24 h. pαIgM: cells were “pulsed” for 30 min at 4°C with α-IgM, washed and then treated with α-CD40 or LPS for 24 h. Shown are mean values of *Prdm1* RNA reads (RPKM) from two assays. **(B)** Splenic B cells from wild-type (WT) mice (open columns) or *caNfatc1/*α*A* × *Cd23-cre* mice (black) were treated for 24–72 h with LPS. Real-time PCR assays for detecting *Prdm1* RNA levels. Mean values of three assays are shown. **(C,D)** WT and *caNfatc1/*α*A* × *Cd23-cre* mice were immunized with NP-KLH **(C)** or NP-Ficoll **(D)** for 21 days, and the serum levels of immunoglobulins were determined in ELISAs. One dot and square indicates one mouse, respectively.

Stimulation of murine splenic B cells with α-IgM resulted in a more than fivefold decrease of *Prdm1* RNA reads within 24 h whereas α-CD40 or LPS treatment increased the number of reads approximately twofold and more than fivefold, respectively (Figure 4A). Prolongation of LPS treatment led to a strong increase of *Prdm1* RNA levels within 48–72 h (Figure 4B) and in massive production of IgM, a typical sign of differentiation of B cells to plasmablasts *in vitro*. However, when splenic B cells were pre-treated with α-IgM followed by LPS, or co-stimulated with α-IgM and LPS, a reduction in *Prdm1* RNA levels and plasmablast formation was observed (16). To show whether the suppression of plasmablast differentiation by α-IgM and, therefore, BCR signals—which induce NFATc1/αA—are indeed due to NFATc1/αA induction we investigated plasmablast differentiation of splenic B cells from tg mice which express a ca version of NFATc1/αA (caNFATc1/αA) from the *Rosa26* locus upon cre-mediated deletion of a STOP sequence. Upon crossing those mice with *Cd23-cre* mice, the splenic B cells of *caNFATc1/*α*A* × *Cd23-cre* mice express caNFATc1/αA (Figure S4 in Supplementary Material), and upon treatment of cells with LPS for 1–3 days *in vitro* a strong reduction in *Prdm1* RNA expression was observed (Figure 4B). When such *caNFATc1/*α*A* × *Cd23-cre* mice were immunized with NP-KLH, a T cell dependent antigen, the secretion of IgG2a Abs into the serum was abolished and those of IgG1 and IgG2b markedly impaired, similar to IgM Ab secretion (Figure 4C). Immunization with NP-Ficoll, a T cell-independent antigen, resulted in a strong decrease in IgM, IgG2b, and IgG3 secretion, and a more moderate in IgG2a secretion (Figure 4D). This suggests that overexpressing caNFATc1/αA suppresses plasma cell formation *in vivo*.

**Figure 5:**
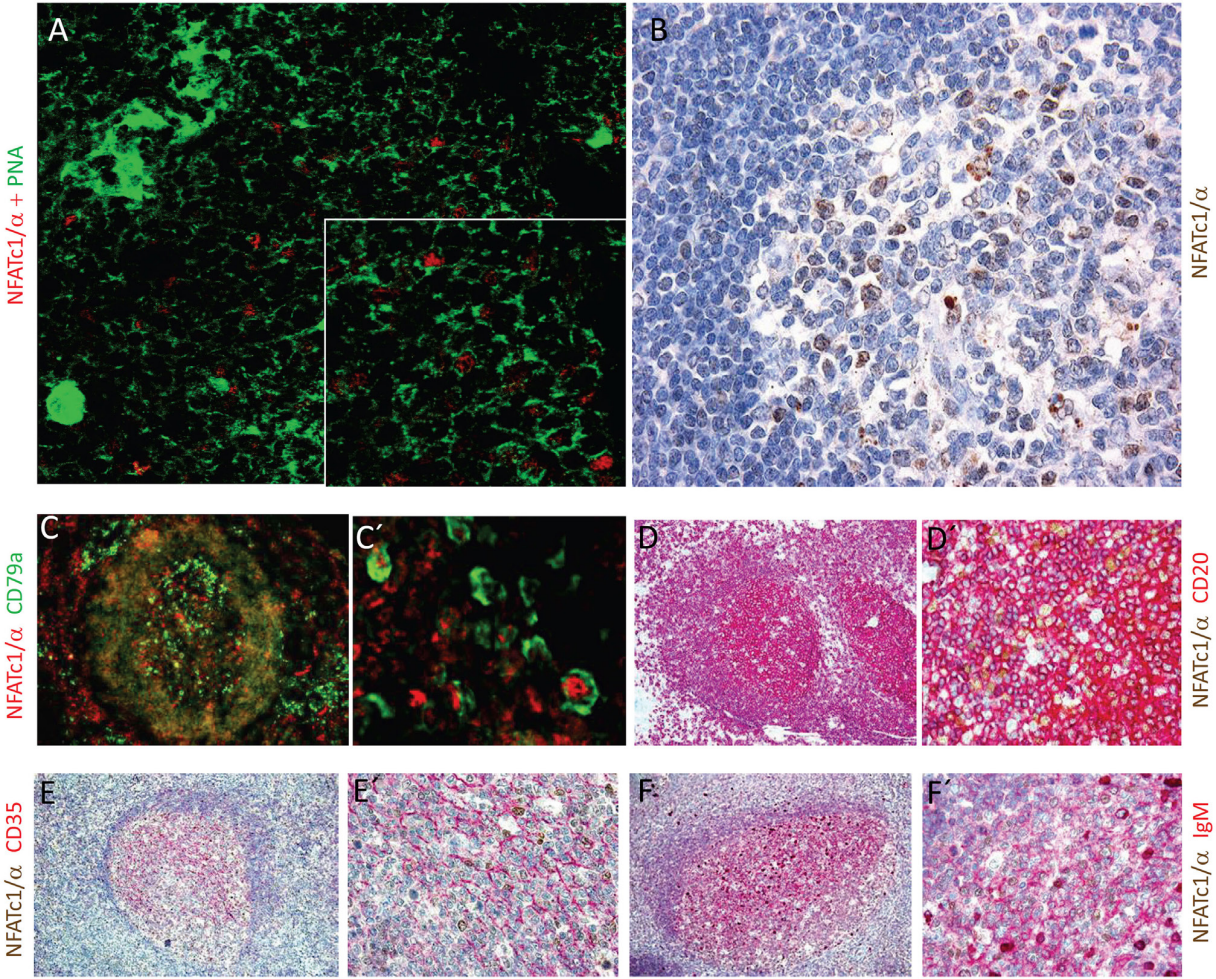
Nuclear expression of NFATc1/αA in germinal center (GC) B cells. **(A)** Histochemical co-stains of GC cells with peanut agglutinin and an antibody (Ab) raised against the N-terminal α-peptide of NFATc1. A spleen section from a mouse immunized with sheep red blood cells for 7 days is shown. Magnification, 20-fold, and insert 40-fold. **(B–F)** Cross sections of tonsils from tonsilitis patients. **(B)** Co-stains of hematoxylin/eosin and an Ab specific for the NFATc1-α peptide. **(C–F)** Co-stains of NFATc1-α-specific Ab with Abs specific for CD79a **(C,C′)**, CD20 **(D,D′)**, CD35 **(E,E′)**, and IgM **(F,F′)** and at various magnifications (C vs. C′ etc.) are shown. Magnifications are 20-fold **(B)**, 10-fold **(C–F)**, 40-fold **(D′–F′)**, or 50-fold **(C′)**.

The NFATc1/αA-mediated control of genes expressed during the GC reaction and plasma cell formation prompted us to investigate whether NFATc1/αA is indeed expressed in nuclei of GC B cells. To this end, we raised a polyclonal Ab against the N-terminal α-peptide of human NFATc1 spanning 42 amino acids, and in histochemical stains of splenic GCs in mice immunized with sheep red blood cells for 7 days, we detected a strong nuclear staining for NFATc1/αA in numerous GC cells (Figure 5A). Similar results were obtained by α-NFATc1/αA staining of human tonsils from patients with chronic tonsillitis showing the nuclear expression of NFATc1/α in numerous GC cells (Figure 5B). By double-staining with Abs directed against CD79a or CD20, several of these GC cells could be identified as B cells (Figures 5C,D). Double-stainings with an Ab specific for CD35, a marker of follicular dendritic cells, show the nuclear localization of NFATc1/αA in GC cells of light zone (Figure 5E), and further double-stainings with Abs directed against various Ig proteins indicated a co-expression of NFATc1/αA with IgM (Figure 5F), but not with IgG or IgA proteins (not shown).

## Discussion

The data presented here and those of a former report (16) show that in primary splenic B lymphocytes BCR-stimulation leads to rapid but transient appearance of nuclear NFAT complexes that consist predominantly of NFATc1. Albeit less reflected at the level of *Nfatc1* transcription, persistent BCR and co-stimulatory signals induce high nuclear NFATc1/αA concentrations which, supported by the NFATc1-mediated auto-regulation of the *Nfatc1* P1 promoter (7), remain high for days. By contrast, BCR signals alone lead to accumulation of cytosolic NFATc1/αA that is kept in cytosol without further (co-) induction. Although primary B cells stimulated by α-IgM alone proliferate and accumulate high levels of cytosolic NFATc1/αA, they die after 3–4 days of *in vitro* culture.

Published data on the strong expression of NFATc1 in follicular T_FH_ cells (32) and our data indicate that NFATc1 is highly expressed in subsets of GC cells. The data presented here show that NFATc1/αA occurs in nuclei of numerous centrocytes within the light zone of a GC that are closely associated with the network of follicular dendritic cells. On the surface of follicular dendritic cells intact antigens are presented which, thereby, help to select GC B cells (2). It is likely that the contact between follicular dendritic cells and centrocytic B cells leads to the high levels of nuclear NFATc1/αA induction which help B cells to survive. The positive effect of NFATc1/αA on Bcl-6 and AID expression suggests that, in analogy to T cells (33), NFAT factors and, in particular, NFATc1/αA support the maturation of GC B cells to memory B cells. The occurrence of nuclear NFATc1/αA in IgM^+^ centrocytes suggests a role for NFATc1/αA in the survival of IgM^+^ memory B cells that constitute a substantial part of memory B cells upon primary immunization with complex antigens (34, 35) but further experiments are necessary to substantiate this view. In contrast to genes that control the GC reaction, NFATc1/αA suppresses the expression of the *Prdm1* gene encoding the key factor of plasma cell development, Blimp-1. The binding of bio-tagged NFATc1/αA to a regulatory region of the *Prdm1* gene indicates *Prdm1* as a direct target of NFATc1/αA which, when overexpressed in centrocytic B cells impairs plasma cell formation.

## Ethics Statement

Informed consents were obtained from patients for this study. Animal experiments were performed according to project licenses (Nr.55.2-2531.01-80/10 and 169), which are approved and controlled by the Regierung von Unterfranken, Würzburg.

## Author Contributions

KM, RR, DP, SK-H, KT, and NM performed experiments; VE provided important material; EK designed and performed experiments, analyzed data, and supported the preparation of the manuscript; ES led the investigation and wrote the manuscript along with KM.

## Conflict of Interest Statement

The authors declare that the research was conducted in the absence of any commercial or financial relationships that could be construed as a potential conflict of interest.
