# Supplementary material for: Induction of Short NFATc1/αA Isoform Interferes with Peripheral B Cell Differentiation
Source: Front Immunol. 2018 Jan 24;9:32. doi: 10.3389/fimmu.2018.00032 (PMC5787671; doi:10.3389/fimmu.2018.00032)
Supplement: Supplementary file 1 [file Presentation_1.PDF]

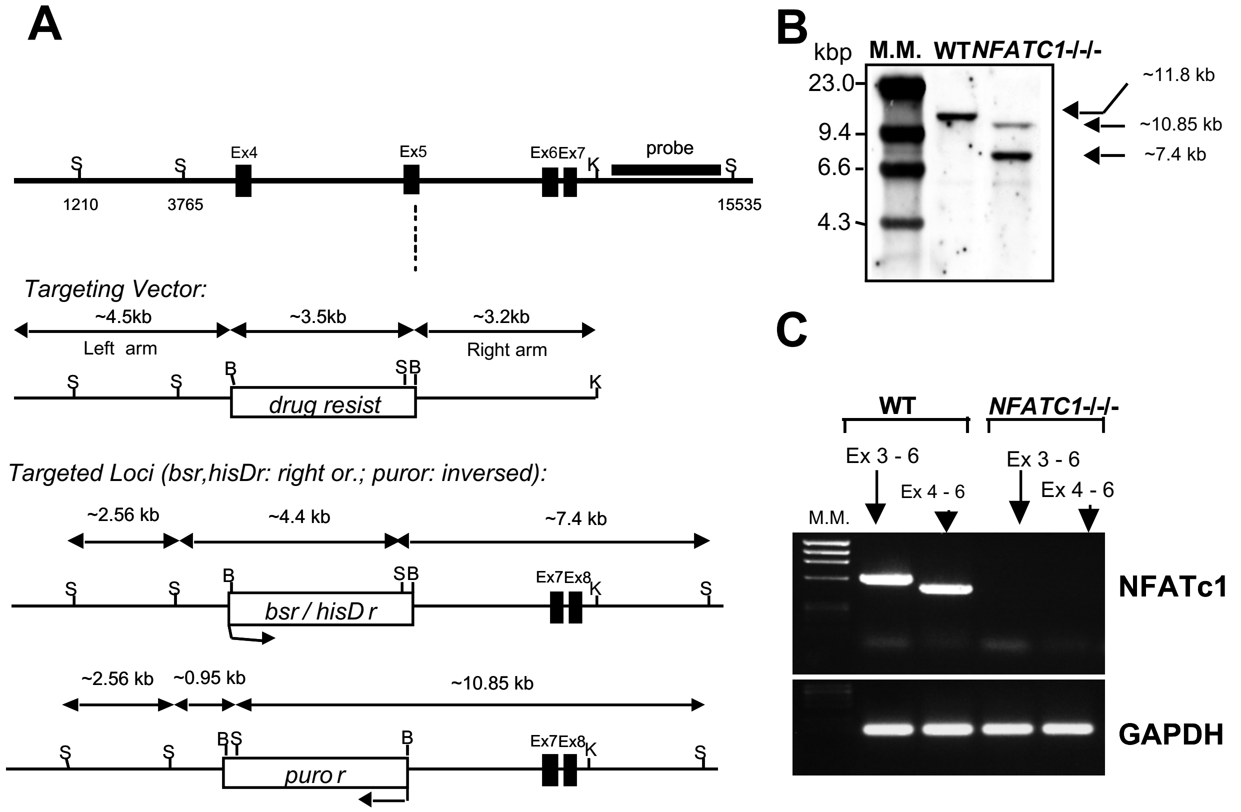

Figure S1. Inactivation of the chicken *NFATC1* gene in DT40 B lymphoma cells. (A) Scheme of targeting strategy for the inactivation of *NFATC1* gene. Above, a portion of the chromosomal chicken *NFATC1* locus is shown including exons 4, 5, 6 and 7. Restriction sites are indicated for the cleavage by Sac I (S), Bam HI (B) and Kpn I (K). The probe which was used for the Southern blot in (B) is shown by a black bar. Targeting vectors were constructed by the assembly of a resistance gene (blasticidin S, bsr; histidinol D, hisDr; puromycin, puror) with two chromosomal *NFATC1* DNA segments of 4.5 kb and 3.2 kb, respectively. Site specific recombination resulted in the appearance of three targeted loci containing either the *bsr* and *hisDr* genes in the orientation of transcription, or the *puror* gene in the reversed orientation. (B) Southern blot showing the appearance of Sac I fragments of 10.85 kb upon site-specific integration of puro r vector, and of 7.4 kb upon integration of bs r or hisD r vectors into the *NFATC1* locus. (C) PCR assays for the detection of *NFATC1* RNA in wild type (WT) and *NFATC1*-deficient (-/-/-) DT 40 cells. Two different primer pairs were used for detecting *NFATC1* RNAs from exons 3 and 6 or exons 4 and 6, respectively. M.M., molecular marker DNA.

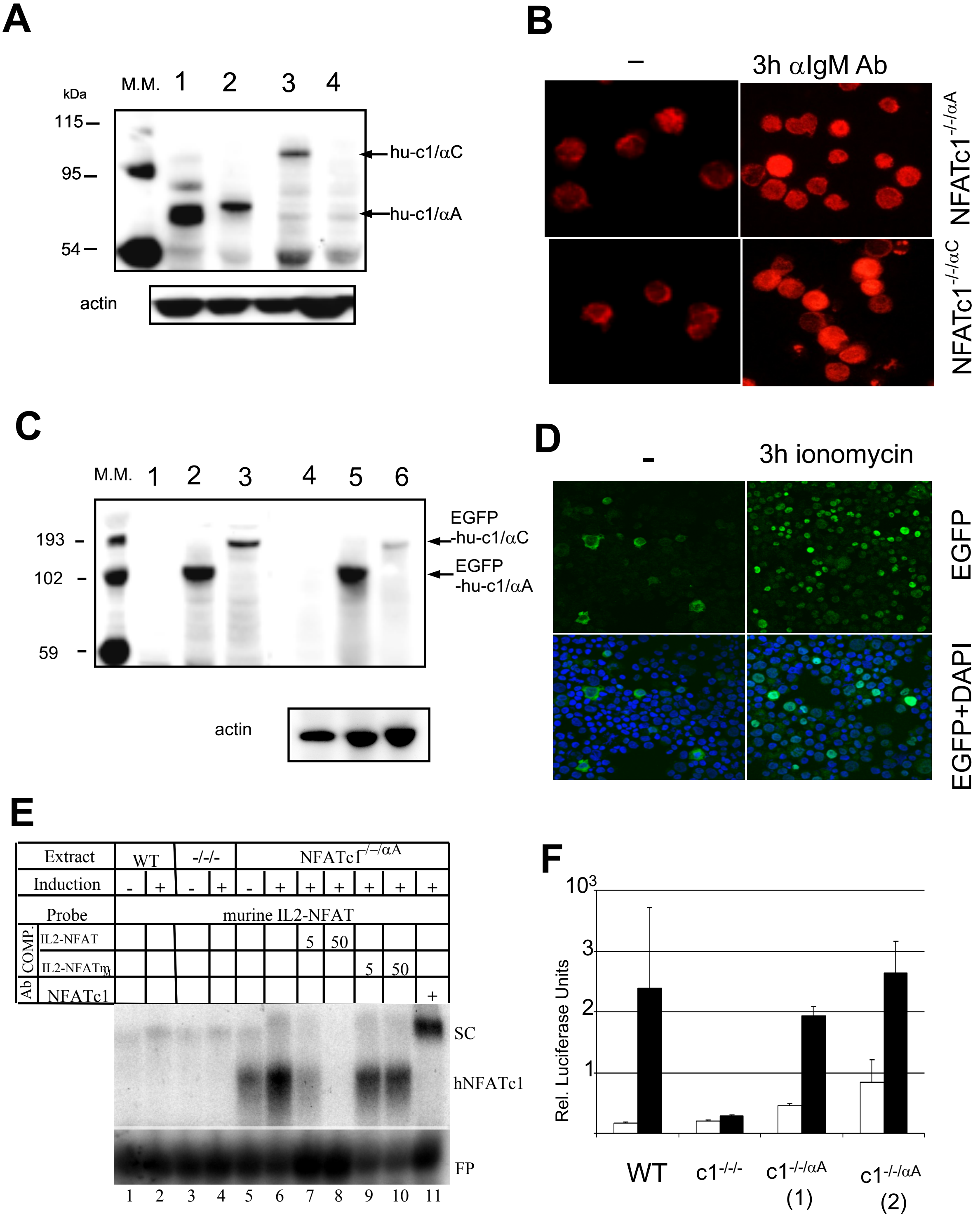

Figure S2. Introduction and ectopic expression of human NFATc1/αA and NFATc1/αC in *NFATC1$^{-/-/-}$* DT40 cells. (A) Western blot showing the expression of human NFATc1/αA or αC proteins tagged with a myc epitope in NFATc1$^{-/-/-}$ DT40 cells. Cells were stably transfected with pcDNA3.1-based neomycin-vectors which express either NFATc1/αA-myc (lanes 1+2) or NFATc1/αC-myc (lane 3). In lane 4, protein from non-transfected DT40 cells was fractionated. The blot was incubated with the mAb 9E10 directed against an epitope of c-Myc. M.M., molecular marker DNA. (B) Detection of nuclear translocation of human NFATc1 proteins upon stimulation of DT40 cells by αIgM. (C) Expression of chimeric EGFP-NFATc1 proteins in *NFATC1$^{-/-/-}$* DT40 cells. Proteins from cells stably transfected with vectors expressing either EGFP-NFATc1/αA (lanes 2+5) or EGFP-NFATc1/αC (lanes 3+6) were blotted and immunodetected with the 7A6 NFATc1-mAb (lanes 1-3) or an pAb directed against EGFP. In lanes 1+4, proteins were fractionated from non-transfected DT40 cells. (D) Detection of nuclear translocation of chimeric EGFP-NFATc1/αA protein

upon treatment of *NFATC1*$^{-/-\alpha A}$ DT40 cells with 1 mM ionomycin for 3 h. (E) Increase of NFATc1/αA DNA binding in *NFATC1*$^{-/-/\alpha A}$ DT40 cells. ÈMSAs using nuclear proteins from non-induced (-) wild type (WT), *NFATC1*$^{-/-/-}$ and *NFATC1*$^{-/-/\alpha A}$ DT40 cells and from cells induced by T+I for 4 h were performed with the distal NFAT binding from the murine *Il2* promoter as probe (FP, free probe). In lanes 7-10, 5 or 50 ng of a WT or mutated oligo of distal *Il2* NFAT site was used for competition. In lane 11, an NFATc1-specific Ab was added (SC, supershift). (F) Increase of NFAT activity in *NFATC1*$^{-/-/aA}$ DT40 cells. A luciferase reporter gene controlled by two copies of distal NFAT site from the murine *Il2* promoter was tranfected into WT, *NFATC1*$^{-/-/-}$ and two lines of *NFATC1*$^{-/-/\alpha A}$ DT40 cells. 24 h later, the cells were left untreated treated (open columns) or treated for another 24 h by T+I (black columns).

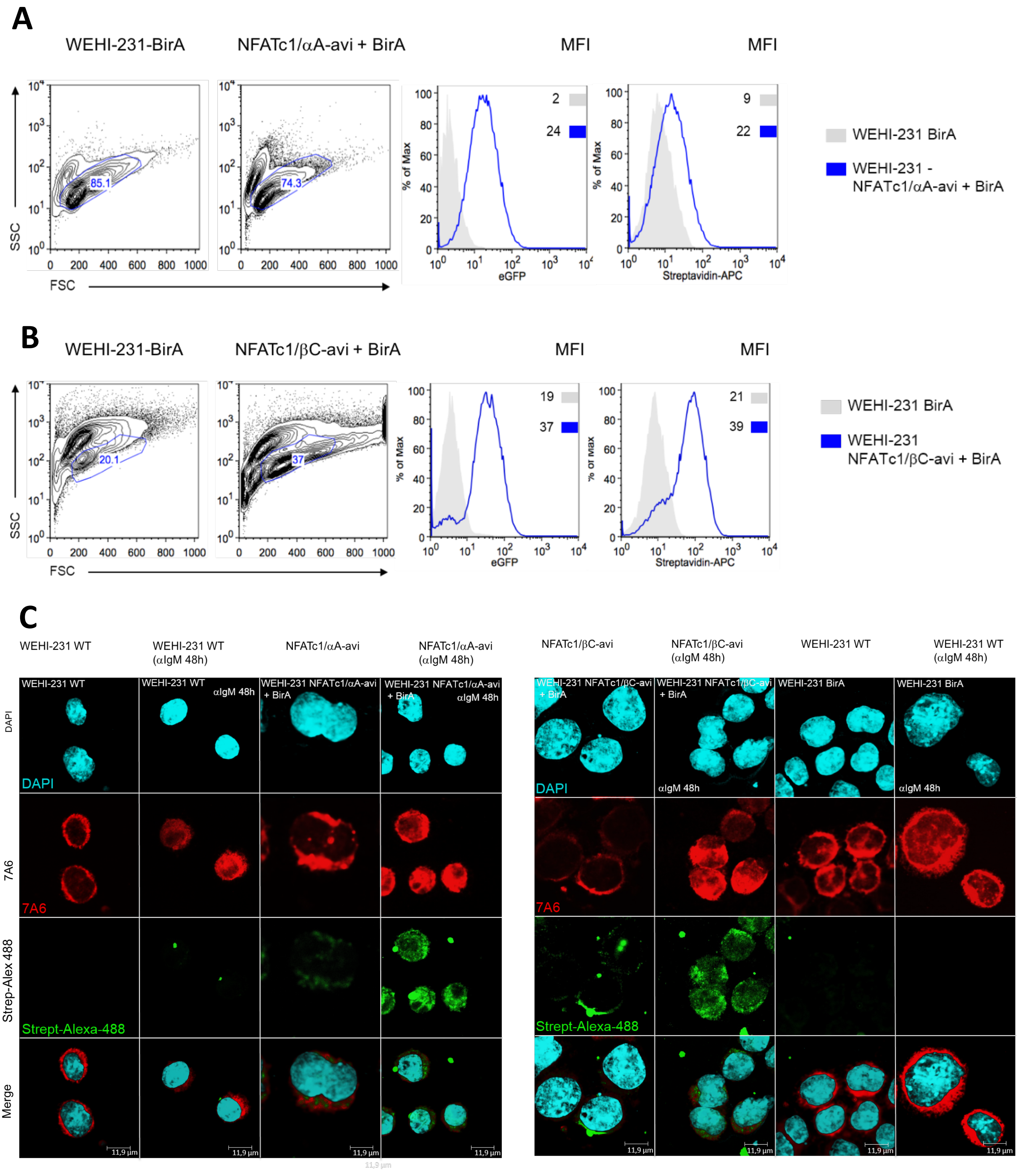

Figure S3. Analysis of WEHI-231 B cells (over-) expressing NFATc1/αA or NFATc1/βC. (A) WEHI-231 cells expressing BirA alone, or WEHI cells expressing NFATc1/αA-avi + BirA were intracellularly stained with streptavidin-APC. Living cells were gated (in SSC and FSC dot-plots), and living cells were measured for eGFP and APC expression. (B) WEHI-231 BirA and WEHI-231 NFATc1/βC-avi + BirA cells were intracellularly stained with streptavidin-APC. Living cells were analyzed for eGFP and APC expression. (C) WEHI 231 WT cells or WEHI cells over-expressing NFATc1/αA-avi (and BirA) (left panel), or NFATc1/βC-avi (right) were harvested by cytospin, fixed on glass slides and stained with an Ab directed against NFATc1 (+ a secondary fluorophore-coupled Ab) or with streptavidin-alexa-fluor 488. The cells were also stained with DAPI.

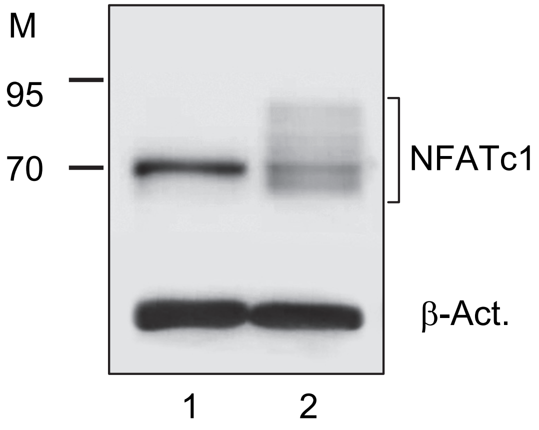

Figure S4. Expression of a constitutive active version of NFATc1/αA in splenic B cells from *caNfatc1/αA x Cd23-cre* mice. Western blot of whole cellular protein from freshly prepared splenic B cells of a *caNfatc1/αA x Cd23-cre* mouse (lane 1) and a wild type mouse (lane 2). M, molecular weight marker.
